# Optimizing NV magnetometry for Magnetoneurography and Magnetomyography applications

**DOI:** 10.3389/fnins.2022.1034391

**Published:** 2023-01-12

**Authors:** Chen Zhang, Jixing Zhang, Matthias Widmann, Magnus Benke, Michael Kübler, Durga Dasari, Thomas Klotz, Leonardo Gizzi, Oliver Röhrle, Philipp Brenner, Jörg Wrachtrup

**Affiliations:** ^1^Institute of Physics, University of Stuttgart, Stuttgart, Germany; ^2^Quantum Technology R&D Center, Beijing Automation Control Equipment Institute, Beijing, China; ^3^Institute for Modelling and Simulation of Biomechanical Systems, University of Stuttgart, Stuttgart, Germany; ^4^Department of Biomechatronic Systems, Fraunhofer Institute for Manufacturing Engineering and Automation IPA, Stuttgart, Germany; ^5^ZEISS Innovation Hub @ KIT, Eggenstein-Leopoldshafen, Germany

**Keywords:** nitrogen-vacancy center, magnetometer, MMG, MNG, sensitivity, bandwidth, dynamic range

## Abstract

Magnetometers based on color centers in diamond are setting new frontiers for sensing capabilities due to their combined extraordinary performances in sensitivity, bandwidth, dynamic range, and spatial resolution, with stable operability in a wide range of conditions ranging from room to low temperatures. This has allowed for its wide range of applications, from biology and chemical studies to industrial applications. Among the many, sensing of bio-magnetic fields from muscular and neurophysiology has been one of the most attractive applications for NV magnetometry due to its compact and proximal sensing capability. Although SQUID magnetometers and optically pumped magnetometers (OPM) have made huge progress in Magnetomyography (MMG) and Magnetoneurography (MNG), exploring the same with NV magnetometry is scant at best. Given the room temperature operability and gradiometric applications of the NV magnetometer, it could be highly sensitive in the $\text{pT}/\sqrt{\text{Hz}}$-range even without magnetic shielding, bringing it close to industrial applications. The presented work here elaborates on the performance metrics of these magnetometers to the state-of-the-art techniques by analyzing the sensitivity, dynamic range, and bandwidth, and discusses the potential benefits of using NV magnetometers for MMG and MNG applications.

## 1. Introduction

Nowadays, with the advances in magnetometry techniques, especially the development of high-sensitive quantum magnetometers, bio-magnetic field measurements are more and more expected to be used routinely as clinical diagnostic tools. The origin of bio-magnetic fields is electrical currents inside the human body which are ultimately caused by the electrophysiological behavior of excitable cells (Malmivuo et al., 1995; Kandel et al., 2000), for example, neurons or muscle cells. One specific bio-magnetic field sensing modality is magnetocardiography (MCG), measuring the magnetic field stemming from the electric excitation of the heart (Fenici et al., 2005). The MCG is associated with the strongest bio-magnetic fields occurring in the human body, i.e., in the range of nT. Weaker bio-magnetic fields in the range of pT to nT are generated by muscle activities. Cohen and Givler (1972) formally proposed magnetomyography (MMG) to detect magnetic fields induced by skeletal muscles. Among the smallest bio-magnetic fields in the range of fT to pT are signals generated by the brain or peripheral nerves. Magnetoencephalography (MEG) and magnetoneurography (MNG) are intensively investigated as the non-contacting alternatives to electroencephalography (EEG) for understanding the function of the brain (Supek and Aine, 2016). To detect such small bio-magnetic fields, highly sensitive magnetometers are required to build up bio-magnetography systems. State-of-the-art magnetometers with fT sensitivity include superconducting quantum interference devices (SQUID) and optically pumped magnetometers (OPM). Because of the cryogenic requirement, systems based on SQUID magnetometers are too expensive for maintenance which consumes liquid helium regularly. SQUID systems also lack flexibility, i.e., are not portable, as they need to be operated at cryogenic temperatures. As a result, SQUID-based systems are still not widely used for diagnostics. Although cheaper and more flexible than SQUID, the dynamic range of OPMs with fT sensitivity is so small that magnetic shielding is essential in the applications, which leads to an extra cost. In applications, magnetic shielding is usually needed for any type of bio-magnetic field measurement to suppress environmental noises. Otherwise, array configurations are required for improving the signal noise ratio (Vrba and Robinson, 2002; Clancy et al., 2021). One of the key parameters for establishing array configurations is the dynamic range, which is still progressing for the OPMs (Robinson et al., 2022). Therefore, the OPM-based bio-magnetic fields detecting systems are still in an early stage. Another important characteristic for such applications is the potential for the miniaturization of these sensors. This is also related to the spatial resolution that can be achieved with the sensors. It is difficult for the current OPMs to be further integrated into a sub-cm dimension due to the constraints imposed by sensor heating and its dissipation. On the other hand, fluxgate sensors and magnetoresistive sensors are currently being explored for bio-magnetic field sensing due to their remarkable ability for miniaturization. However, these sensors have a trade-off between the sensitivity and the sensor volume, and the miniaturized sensors usually have a sensitivity in the range of a few nT—50 pT (Chaves et al., 2007; Ripka and Janosek, 2010; Liou et al., 2011; Cubells-Beltrán et al., 2016; Zuo et al., 2020). The optomechanical magnetometer is another type of sensor that is very suitable for miniaturization, but a sensitivity of $<1\text{ nT}/\sqrt{\text{Hz}}$ can only be achieved in the MHz range (Li et al., 2018a,b, 2020). As a summary, it is still expected to find a technology that can well combine miniaturization with $\text{fT}/\sqrt{\text{Hz}}$ to $\text{pT}/\sqrt{\text{Hz}}$ sensitivity.

The recent progress in magnetometers based on negatively charged nitrogen-vacancy (NV) centers in diamonds provides another option for bio-magnetic field detection. One of the most attractive advantages of the NV magnetometers is the high sensitivity per volume at room temperature, which indicates that the sensors can achieve high sensitivity with a very small sensing volume (Taylor et al., 2008). This ensures the possibility of developing large-scale high-sensitive sensor arrays for unshielded bio-magnetic sensing applications. The NV magnetometry has shown high sensitivities from pT to sub-pT level with the diamond volume of roughly a hundred μm^3^ (Fescenko et al., 2020; Zhang et al., 2021). In the laboratory, we have developed endoscopic sensor heads with a parabolic lens in millimeters, which is shown in the later text. Unlike SQUIDs that need cryogenic and OPMs that need heating to the vapor cells, NV magnetometers do not need any thermal insulation so that the distance to the sensing target can be reduced. The NV centers are sensitive to thermal fluctuations, leading to measurement errors and reduced sensitivity in magnetometry applications. However, these errors are shown to be suppressed by methods such as the double quantum magnetometry (Mamin et al., 2014; Bauch et al., 2018; Barry et al., 2020). As a result, the shorter sensing distance enhances the detected signal magnitude so that weak bio-signals such as single neuron signals can be detected (Barry et al., 2016). The small sensing volume of NV magnetometers is also expected to be used for gradiometry, which can improve the depth resolution for applications such as MMG. Besides, efforts have been made to enhance the dynamic range of NV magnetometers (Clancy et al., 2021; Zhang et al., 2022), and the dynamic range of NV magnetometers is much larger than the OPMs. Therefore, NV magnetometers can be more promising for developing an unshielded gradiometry system for bio-magnetic field sensing.

In this work, we introduce NV magnetometry with its performance matrix. We present the sensitivity, bandwidth, and dynamic range for the two main principles of NV magnetometry and demonstrate the simulated action potential signal detection with a muscular phantom by the setup. Finally, we discussed the potential application of NV magnetometer and gradiometer for MNG and MMG, expecting unshielded NV-based bio-magnetic field measurements in the future.

## 2. Characteristics of NV magnetometer

### 2.1. Basics of NV magnetometry

**Figure 1:**
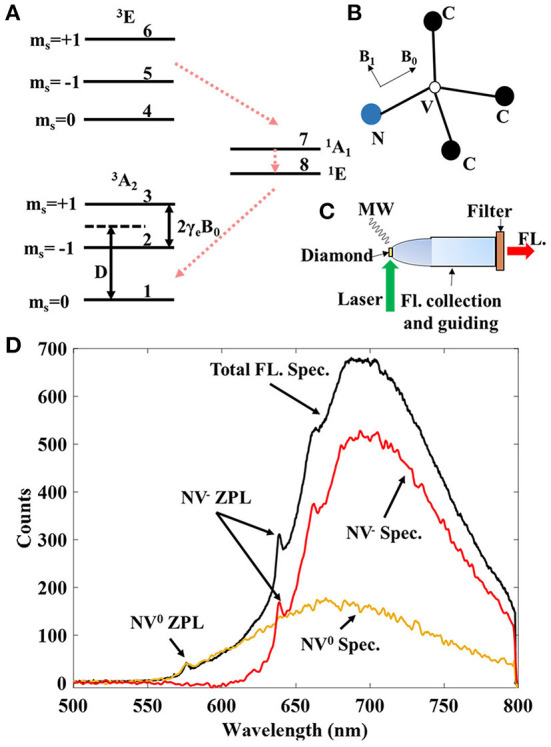
**(A)** Energy level diagram of negatively charged NV center in diamond. The bias field *B*_0_ degenerate the *m*_*s*_ = ±1 states by 2γ_*e*_*B*_0_. The zero-field splitting is *D* ≈ 2.87 GHz. The numbers are used to label the different levels. **(B)** Structure of NV center in diamond. The applied *B*_0_ field is parallel to the N-V orientation, and the applied driving field *B*_1_ is supposed to be perpendicular to the N-V axis. **(C)** A typical setup of the NV magnetometer. The green laser is illuminated on the diamond, which is assembled with the optics to collect and guide the fluorescences for detection. The microwave field is used for resonant driving of the spin states. **(D)** Fluorescence spectrum (the highest black line) emitted by NV center ensembles in a 0.5^3^mm^3^ diamond. The signals from the two different charge states, i.e., NV^−^ (the middle red line) and NV^0^ (the lowest yellow line) centers, are estimated in the figure.

As shown in Figure 1B, the NV center is a substituted nitrogen atom connected with a vacancy. With a captured electron, negatively charged NV center energy level structure can be simplified to an *S* = 1 triplet ground state (labeled as 1, 2, 3), the corresponding triplet excited state (labeled as 4, 5, 6), and two singlet states (labeled as 7, 8), as depicted in Figure 1A. A zero-field splitting (ZFL) *D* = 2.87 GHz in the ground state between *m*_*s*_ = ±1 and *m*_*s*_ = 0 along the N-V axis defines the quantization axis, which naturally makes the NV center in diamond a vector magnetometer. The energy gap between the ground state and the excited state is 1.945 eV (637 nm). The excited state can directly decay to the ground state with the same projection quantum number and emit a 650–800 nm photon. Otherwise, it can also decay through a dark path intermediated by singlets, and the spin projection quantum number is changed from excited state *m*_*s*_ = ±1 to ground state *m*_*s*_ = 0 and cause a strong polarization to *m*_*s*_ = 0, which is enabled by spin-orbit coupling and phonon effect. This decay path also leads to the fluorescence reduction of the excited state *m*_*s*_ = ±1, resulting in an efficient optical readout for the spin state. The dashed red arrow lines indicate the second decay path. The sensing principle of NV magnetometry is based on the Zeeman shift of the substates 2 and 3.

Figure 1C shows a typical NV probe that we use in experiments. The green laser (532 nm) is illuminated directly onto the diamond for excitation. The fluorescence emitted from the diamond is then collected by the parabolic lens and guided by the optic pipe to the photo-detection end. The long pass filter (LPF) blocks the laser line and some irrelevant fluorescence to increase the contrast. The microwave field is generated by the nearby loop antenna for driving the spins. This setup ensures that the diamond can be close to the sensing target. The optics are designed to collect fluorescence as much as possible. However, the way of laser illumination is still not ideal for endoscopic applications. One solution could be to use a dichroic beam splitter to have the laser illumination from the detection end. The cut-on wavelength of the LPF is also important for improving sensitivity by increasing the fluorescence contrast. This is due to the existence of the neutral NV centers, which is further elaborated in Figure 1D.

It is almost inevitable to generate neutral NV (NV^0^) centers in the creation of NV^−^ centers in diamond. The two different types of color centers emit fluorescence with similar wavelengths under green laser excitation. In Figure 1D, we show the measured total fluorescence spectrum from the diamond and the estimated fluorescence spectra of NV^−^ and NV^0^ centers. The zero-phonon-line (ZPL) of NV^0^ is 575 nm, while the ZPL of NV^−^ is 637 nm. With the two ZPLs, the spectra of NV^−^ and NV^0^ centers are estimated by the microwave-assisted spectroscopy method (Craik et al., 2020). The method distinguishes the fluorescence spectrum of NV^−^ from the total fluorescence spectrum, i.e., by measuring the signal difference both in the presence and absence of the MW driving field. Therefore, the fluorescence wavelengths of NV^0^ are shorter than that of NV^−^ centers. Nevertheless, there are coincidental photons due to the phonon sideband, and the signal contrast is reduced if the LPF is not optimized. To optimize the LPF, we estimate the charge state ratio based on the microwave-assisted spectroscopy method (Craik et al., 2020). The ZPLs of NV centers are used as the indicators for the estimation, of which the result is shown in Figure 1D. Then, the LPF can be optimized to exclude most of the photons emitted from NV^0^ but keep the NV^−^ signal as much as possible for high sensitivity. Based on the optically optimized setup, we investigate the magnetometry schemes to explore the possible specifications of the NV magnetometer.

**Figure 2:**
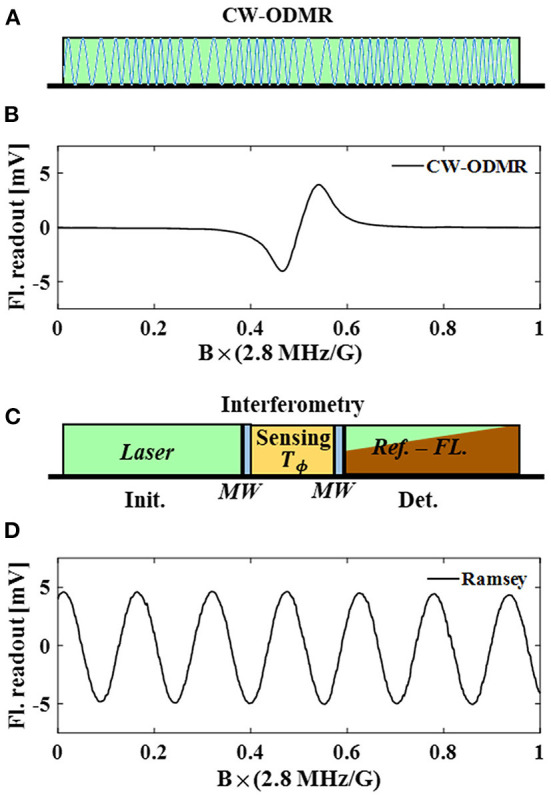
**(A)** CW-ODMR method uses continuous wave laser pumping and MW field driving. The MW field is modulated in frequency or phase to get a modulated fluorescence signal. **(B)** CW-ODMR spectrum demodulated by the lock-in amplifier. MW frequency shift is used to simulate the line shift induced by an external field. *B* × (2.8 MHz/G) is used as the description of the horizontal axis to show the relationship between the external field (in Gauss) and the line shift (in MHz). **(C)** Most of the interferometry methods consists of three parts, i.e., initialization, sensing, and detection. In the initialization part, a green laser (532 nm) is used to polarize spin states into *m*_*s*_ = 0, regardless of the fluorescence emitted from the diamond. In the sensing part, the two blue blocks represent two MW pulses in a Ramsey sequence, and the yellow block depicts the magnetic field sensing time. The Ramsey sequence can be replaced by different interferometry sequences to measure dc/ac magnetic fields. In the detection part, fluorescences are collected to readout the spin population which indicates the spin-detected magnetic field information. The wedge red indicates that the fluorescence drops at the beginning of the detection window and continuously increases due to the repolarization induced by the detection-laser pulse. **(D)** The sensor response of Ramsey measurement to the MW frequency shift, which is equivalent to the response to the magnetic field.

Generally, there are two types of magnetometry schemes for the NV sensor. One is based on the ODMR spectrum, in which the shifts of the resonant lines detect external magnetic fields (Shin et al., 2012). The other one is based on the interferometry of NV spins, in which the quantum phase accumulated from the interaction between the spins and the external fields is measured to interpret the magnetic fields (Taylor et al., 2008; Barry et al., 2020). In Figure 2A, the illustration describes the scheme of a continuous-wave (CW) ODMR detection, in which laser excitation and microwave driving are continuously applied. By modulating the microwave, e.g., frequency modulation (FM) and phase modulation (PM), we acquire the ODMR spectrum from a lock-in amplifier when sweeping the microwave frequency, as shown in Figure 2B. There is a bias magnetic field of 10 Gauss applied along the [111] direction of the diamond. Since the shift of the microwave frequency is equivalent to the change of the magnetic field multiplying the electron gyromagnetic ratio γ_*e*_ = 2.8 MHz/Gauss, the lock-in detected spectrum can be used to indicate the characterizations of the magnetic field sensing. Figure 2C illustrates the typical Ramsey scheme as an example of the interferometry schemes. An interferometry measurement comprises three parts, i.e., initialization, sensing with the spin manipulation sequence, and detection. We denote the effective sensing time as *T*_ϕ_. This interaction time should be smaller than the spin coherence time, and it determines the magnetic field sensing response together with the spin manipulation sequence. The Ramsey sequence can be used for dc field sensing, and many more sequences, e.g., dynamical decoupling sequences, are used for ac field sensing. In Figure 2D, we present the fluorescence readout of the Ramsey sequence with the sweeping of the microwave frequency. The measurement is also carried out with a bias field of 10 Gauss. The response shows the interferometric result with the horizontal axis the same as Figure 2B, indicating the same response to the external magnetic field. In Section 2.2, we elaborate on the optimization of NV magnetometry for both CW-ODMR and the interferometry schemes.

### 2.2. Specifications of NV magnetometry

#### 2.2.1. Sensitivity

The sensitivity limit of a magnetometer can be derived based on the definition

(1) $$\begin{array}{l} \eta =\frac{\sigma \sqrt{t}}{\text{d}S/\text{d}B}, \end{array}$$

where σ is the stand deviation or the noise level of the measured signal, *t* is the measurement time, and d*S*/d*B* is the scalar factor of the measured signal to the magnetic field. For NV magnetometry, the intrinsic noise usually is tracked to the photon shot noise limit. Therefore, $\sigma \sqrt{t}=\sqrt{R}$, where R is the fluorescence photon detection rate.

In the CW-ODMR scheme, d*S*/d*B* is determined by the line profile of the detected spectrum, from which we can get

(2) $$\begin{array}{l} \eta \approx P\frac{\Delta \nu }{\gamma_{e}C\sqrt{R}}, \end{array}$$

where Δν is the linewidth of the spectrum, C is the signal contrast, P is a factor related to the line shape, and γ_*e*_ is the gyromagnetic ratio of electron spin. The linewidth of NV ensembles not only depends on the power broadening but also on the inhomogeneity of the laser beam, MW field, and magnetic field bias. In most devices and setups, such inhomogeneities are static and can be covered by the experimentally measured linewidth and contrast. As a result, the laser power and MW power are still the two remained parameters that need to be optimized in the CW-ODMR sensing scheme. The optical saturation parameter *s* = *P*_*opt*_/*P*_*sat*_ is used to represent the laser pumping effect, where *P*_*opt*_ is the applied laser power, and *P*_*sat*_ is the saturation power. The Rabi frequency Ω_*R*_ is used to represent the square root of MW power. It should be pointed out that when the optimized Rabi frequency is low, a full Rabi oscillation possibly not exists, but the corresponding CW-ODMR spectrum exists. With the measured contrast parameter and the coherence property of the NV ensembles, i.e., the longitude relaxation rate Γ_1_ = 1/*T*_1_ and the transverse relaxation rate $\Gamma_{2}^{*}=1/T_{2}^{*}$, optimization of the parameters is reproduced according to Wolf (2018) Zhang et al. (2021), and Dréau et al. (2011). Additionally, we vary $T_{2}^{*}$ and *T*_1_ to see how the optimized parameters change with the coherence times.

**Figure 3:**
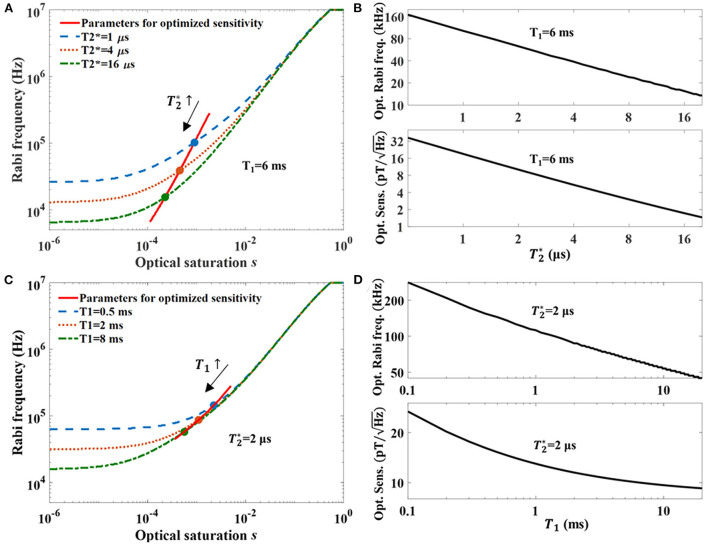
**(A)** Optimization of parameters for the sensitivity of CW-ODMR NV-magnetometry. The three curves (dash, dot, and dash-dot) indicate the Rabi frequencies for the optimized sensitivities with different laser powers. The different curves use different $T_{2}^{*}$ for the calculations. The solid line plots both the laser powers and the Rabi frequencies of the optimized sensitivities for different $T_{2}^{*}$, and the dots are the crossing spots with the other three curves. **(B)** The upper graph shows that the optimal Rabi frequency is logarithmically linear to the $T_{2}^{*}$ of the diamond. The lower graph shows that the calculated optimized sensitivity is also logarithmically linear to the $T_{2}^{*}$. **(C)** Optimization of parameters for sensitivities of diamonds with different *T*_1_. The curves for different *T*_1_ are calculated with $T_{2}^{*}=2\mu s$. The solid line indicates the parameters for optimized sensitivity with different *T*_1_. **(D)** The upper graph shows the relationship of the optimal Rabi frequency for different *T*_1_, and the lower graph shows the best sensitivities that can be achieved by the diamonds with different *T*_1_.

In Figure 3A, *T*_1_ = 6 ms (see in the Supplementary Figure 1) is fixed to see the optimal parameters for different $T_{2}^{*}$. The Rabi frequency is optimized with the given saturation parameter *s* and $T_{2}^{*}$ as the curves shown in the figure. Generally, smaller MW power is required for a longer $T_{2}^{*}$. Further, we calculate the optimized parameters *s* and Ω_*R*_ for the best sensitivity that can be achieved with the given $T_{2}^{*}$. The effects introduced by the inhomogeneities of the laser illumination and the MW field are included in the experimentally measured $T_{2}^{*}$ and Ramsey contrast. The solid line shows the changing of the optimized parameters, and both the required laser power and the MW power are lower with longer $T_{2}^{*}$. Figure 3B shows the relationship between the optimized Ω_*R*_ and $T_{2}^{*}$, and the relationship between the sensitivity and $T_{2}^{*}$. Both the results decrease logarithmically with the increase of the $T_{2}^{*}$. $T_{2}^{*}=8.5\text{ μs}$ was reported and found as one of the longest $T_{2}^{*}$ in experiments (Zhang et al., 2021), and the calculated sensitivity that can be achieved is about $2.9\text{ pT}/\sqrt{\text{Hz}}$. It is also the $T_{2}^{*}$ time we get in this work with the same (0.5 mm)^3^ diamond for demonstrating the NV magnetometry performances (coherence measurements see in the Supplementary Figure 1). Here we note that neither the hyperfine line driving (contrast <3C expected) nor the double resonance driving (contrast <2C expected) techniques are considered in the calculation. In experiments, the contrast enhancements due to the two techniques are × 2.67 and × 1.3 (see Supplementary Figure 3), respectively, which can lead to a sensitivity-limit of $0.9\text{ pT}/\sqrt{\text{Hz}}$. However, the experimental sensitivity is still subjected to technical noises, e.g., laser intensity noise, which deteriorates the sensitivity sometimes more than an order (see Supplementary Figure 2c). Similarly, the optimized operation parameters with *T*_1_ varying and $T_{2}^{*}$ fixed as 2 μs are calculated as shown in Figures 3C, D. The value is chosen as a typical $T_{2}^{*}$ that can be achieved by using isotopically purified diamond. The ^12^C nuclear spin usually perform as the major reason for the dephasing of NV center ensembles. Besides that, the electron spin bath, NV-NV interaction, and strain inhomogeneity can all contribute to the dephasing. Both optimal parameters *s* and Ω_*R*_ change with *T*_1_ as they are changing with $T_{2}^{*}$, but are less sensitive to the variation of *T*_1_. The optimized sensitivity varies with *T*_1_ in a non-linear way. With $T_{2}^{*}=2\text{ μs}$, the optimized sensitivity limit can end up as $<10\text{ pT}/\sqrt{\text{Hz}}$ by increasing *T*_1_.

Compared to the CW-ODMR scheme, interferometry sensing schemes can utilize high laser power and MW power for improving sensitivity. In the CW-ODMR scheme, high laser power broadens the linewidth (linewidth narrowing barely happens when the $T_{2}^{*}$ is a few μs long) and reduces the signal contrast, and high MW power also broadens the linewidth and deteriorates the sensitivity of CW-ODMR measurements (Dréau et al., 2011; Jensen et al., 2013). On the other hand, laser and MW do not introduce linewidth broadening to the pulsed schemes because both of them are not applied during the measurement intervals. As a result, the readout photon counting rate can be improved by the high laser power, and the contrast can be improved by the high MW power. Due to these reasons, pulsed schemes, e.g., Ramsey sequence, are expected to be more sensitive than CW-ODMR measurements. It should also be pointed out that the high laser power and MW power may also have negative impacts on measurements and applications. For example, the high power can lead to thermal issues and the radiation of MW potentially damages bio-samples. The issues should be addressed with solutions of, e.g., MW antennas/resonators and metallic housing.

According to the concept of the interferometry schemes, the accumulated quantum phase is measured. Thus, d*S*/d*B* = dϕ/d*B* needs to be determined in Equation (1). For most sequences, switching functions can be applied to describe the effects in which a π pulse switches the constant value between +1 and −1 (Staudacher et al., 2013). For example, the switching function of the Ramsey sequence is *g*(*t*) = 1, *t* ∈ [*t*_0_, *t*_0_ + *T*_ϕ_], where *t*_0_ is the timestamp at the beginning of the sensing period, and *T*_ϕ_ is the sensing interval time as shown in Figure 2C. The switching function of a sequence consisting of *N* number of π pulses is expressed as

(3) $$\begin{array}{l} g(t)=\{\begin{array}{c} +1,\;t\in \left[\frac{(k-1)T_{\phi }}{2N},\frac{kT_{\phi }}{2N}\right],k\;is\;odd,\\ -1,\;t\in \left[\frac{(k-1)T_{\phi }}{2N},\frac{kT_{\phi }}{2N}\right],k\;is\;even. \end{array} \end{array}$$

**Figure 4:**
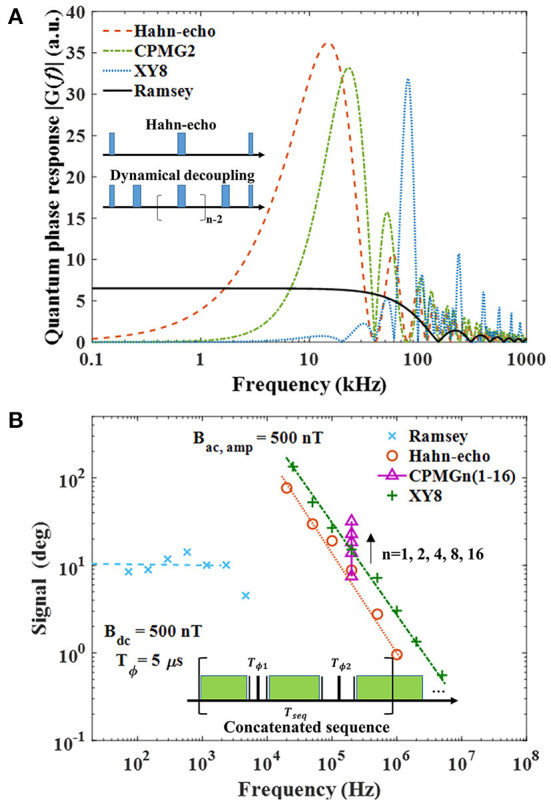
**(A)** Filter functions of different pulsed sequences that can be used in NV-magnetometry. In the calculation of the filter function of the Ramsey sequence, the sensing time *T*_ϕ_ = 6.5μ*s*. In the calculations of the sequences for ac field sensing, i.e., Hahn-echo, CPMG2, and XY8, the sensing time *T*_ϕ_ = 50μ*s*. **(B)** An overview of the measured frequency responses of NV-magnetometry using the different sequences. The QPSD technique is used to read out the field strength by extracting the quantum phase measured by the sequences. The “×” shows the response of the Ramsey sequence, of which the bandwidth is limited by the sampling rate in the experiment. The “+” and the “◦” shows the ac field responses regarding different *T*_ϕ_ when Hahn-echo and XY8 are applied for the measurements. The “△” shows the measurements of a 200 kHz signal by CPMG sequences with different numbers of the π-pulses. One of the primary goals of the NV-magnetometry development is to develop concatenation of the sequences that can ensure a flat frequency response for ac field sensing.

The acquired quantum phase by the pulse sequence can be derived by ϕ = ∫*B*(*t*) · *g*(*t*)d*t*. Here we consider a near dc signal or an ac signal which can be expressed by *B*(*t*) = *Be*^−*i*(ω*t* + φ(ω))^, where *B* is the signal magnitude, ω is the frequency component, and φ(ω) is the initial phase of the signal component. The derivation result of the acquired quantum phase can be simplified as

(4) $$\begin{array}{l} \phi \left(\omega \right)=|G\left(\omega \right)|e^{-i\varphi_{seq}}\cdot \gamma_{e}B\left(\omega \right)e^{-i\varphi \left(\omega \right)}, \end{array}$$

where *G*(ω) is the filter function of the sequence, φ_*seq*_ is the phase response induced by the sequence. In Figure 4A, we demonstrate the quantum phase response of different pulse sequences based on their filter functions. The inset sequences are Hahn-echo and dynamical decoupling sequences (only MW pulses are shown). The Hahn-echo sequence consists of two π/2 pulses and a π pulse in the center, while the dynamical decoupling sequences use multiple π pulses for extending the *T*_2_ time. Usually, the coherence time *T*_2_ for the ac field sensing schemes is much longer than the coherence time $T_{2}^{*}$ for the dc field sensing with Ramsey sequence. Therefore, ac field sensing can be more sensitive than dc field sensing, shown by the signal responses in Figure 4A.

Since the detected fluorescence signal of each sequence can be denoted as s(t)=NCsinϕ, the phase noise is derived as

(5) $$\begin{array}{l} \sigma_{\phi }\approx \frac{\delta F}{NC}=\frac{1}{C\sqrt{N}}, \end{array}$$

where N is the collected photon number per sequence, and $\delta F=\sqrt{N}$ is the photon shot noise. Taking Equations (1), (4), and (5), we get the shot noise limited sensitivity of the interferometry scheme as

(6) $$\begin{array}{l} \eta =\frac{1}{\gamma_{e}|G\left(\omega \right)|C}\sqrt{\frac{T_{seq}}{N}}, \end{array}$$

where *T*_*seq*_ > *T*_ϕ_ is the time length of the sequence. The decoherence term can be added by multiplying the contrast C with the factor $e^{-{\left(T_{\phi }/T_{c}\right)}^{p}}$, where *T*_*c*_ is the coherence time and *p* is the exponential stretching factor.

In order to compare the sensitivity with the CW-ODMR measurement, we calculate with the Ramsey sequence for estimating the dc field sensitivity. The quantum phase response of the dc field to the Ramsey sequence can be derived as

(7) $$\begin{array}{l} \phi \left(\omega \to 0\right)=\gamma_{e}B_{dc}T_{\phi }, \end{array}$$

i.e., *G*(ω → 0) = *T*_ϕ_. Taking *T*_ϕ_ = *T*_*c*_, Equation (6) is rewritten as

(8) $$\begin{array}{l} \eta =\frac{e}{\gamma_{e}T_{c}C_{max}\sqrt{R}}\sqrt{\frac{T_{seq}}{\Delta t}}, \end{array}$$

where N=RΔt, Δ*t* is the fluorescence collection window in the sequence.

Experimentally, we acquire a fluorescence counting rate of $R=4.6\times 10^{15}\text{ Hz}$ and a dephasing time of $T_{c}=T_{2}^{*}=8.5\text{ μs}$. By applying a strong MW field to reduce the MW pulse width, signals from all three hyperfine lines are included, and $C_{max}=0.03$. Neglecting the sensitivity deterioration induced by the sequence $\sqrt{T_{seq}/\Delta t}$, the estimated sensitivity limit $\eta \approx 0.9\text{ pT}/\sqrt{\text{Hz}}$. Double quantum driving can be applied to enhance the sensitivity with a factor of 2 (Mamin et al., 2014; Bauch et al., 2018), where the influence of thermal and strain on the coherence time is suppressed. We estimate the optimized sensitivity as $0.45\text{ pT}/\sqrt{\text{Hz}}$, which is better than the optimized sensitivity of the CW-ODMR measurement. However, the laser power is far lower than the saturation power in the experiments that have the above fluorescence counting rate, and this results in sensitivity deterioration due to the sequence parameters $\sqrt{T_{seq}/\Delta t}$. The laser illumination time in the sequence has to be long enough, e.g., *T*_*seq*_ = 500 μs, for initializing all the spins. Otherwise, contrast is reduced, which also deteriorates the sensitivity. Taking Δ*t* = 10 μs, the sensitivity deterioration due to the sequence can be a factor of 7, i.e., 6.3 $\text{pT}/\sqrt{\text{Hz}}$. An experimentally acquired sensitivity is based on the Ramsey scheme with the $T_{2}^{*}$ is 17 $\text{pT}/\sqrt{\text{Hz}}$. The measured sensitivity is limited by the laser noise at the current stage. Therefore, unlike CW-ODMR, which achieves the optimized sensitivity when the laser power is far below saturation (around 100 mW in our case), interferometry schemes require the laser power near saturation, which is usually a few or even tens of Watts for large NV ensemble. The higher laser power leads to a higher fluorescence counting rate, and sensitivity below 1 $\text{pT}/\sqrt{\text{Hz}}$ can be expected (see estimation in Supplementary Note 1). However, the high sensitivity will be built on high power consumption for the laser saturation, which can easily introduce noise and heating issues.

To briefly summarize the results and discussions, we note that $\text{pT}/\sqrt{\text{Hz}}$ sensitivity can be achieved by optimizing either the ODMR magnetometry or the interferometry magnetometry. Sensitivity can be even at subpicotesla level by using interferometry magnetometry at the cost of very high power consumption.

#### 2.2.2. Bandwidth

One of the most attractive characteristics of NV magnetometry is the wide range of detectable frequencies. The ODMR schemes can be used to detect signals at dc and low frequencies (Barry et al., 2016; Zhang et al., 2021). Meanwhile, signals with frequencies up to GHz can be detected by different pulse schemes (Wolf et al., 2015; Meinel et al., 2021; Wang et al., 2022). Nevertheless, the bandwidth of NV magnetometers usually trades off with different specifications and technical limits. Due to this reason, the bandwidth is not equal to the detectable frequency range. For example, the bandwidth of NV magnetometers is mostly limited by the time constant of the lock-in amplifier (LIA), which is used to avoid flicker noise in the system. In reference (Zhang et al., 2021), the measurement bandwidth is limited to 200 Hz due to the LIA time constant, i.e., the cut-off frequency of the low pass filter in the LIA. However, the intrinsic bandwidth of the CW-ODMR measurement can be much larger. The signal frequency response of the CW-ODMR measurement majorly depends on the laser pumping rate and the longitude relaxation rate Γ_1_ = 1/*T*_1_. The laser pumping tends to reset the spin ensembles so that a higher pumping rate leads to a wider bandwidth but a smaller signal contrast. Similarly, the longitude relaxation tends to deteriorate the spin population so that a higher Γ_1_, i.e., a shorter *T*_1_, also results in a wider bandwidth but a smaller signal contrast. Here, the bandwidth trades with the contrast, which determines the sensitivity. On the other hand, because of the flicker noise in the system, a higher modulation frequency can be expected for the optimal sensitivity with a smaller noise floor but a reduced signal contrast. Thus, the measurement bandwidth can be even larger than the intrinsic bandwidth when the sensitivity is optimized. In our work of Zhang et al. (2021), the intrinsic bandwidth determined by Γ_1_ and *T*_1_ is roughly 1 kHz, while the optimized modulation frequency for lock-in detection is 9 kHz. This means that the measurement bandwidth can approach 9 kHz regardless of the harmonic noises due to the demodulation. Typically, the bandwidth of an NV magnetometer based on CW-ODMR is from dc to a few kHz.

Here in this work, we provide more discussions on the bandwidth based on the interferometry schemes. The filter functions plotted in Figure 4A demonstrate not only the maximum signal responses but also the bandwidths. The calculation of the Ramsey sequence is based on the experimentally optimized *T*_ϕ_ = 6.5 μs, while *T*_ϕ_ = 50 μs is used for the calculation of the other sequences. According to the filter function of the Ramsey sequence, i.e.,

(9) $$\begin{array}{l} G_{0}\left(\omega \right)=T_{\phi }\text{ sinc}\left(\omega T_{\phi }/2\right), \end{array}$$

the bandwidth of the given Ramsey sequence is *BW*_0_ = 1/(π*T*_ϕ_) ≈ 50 kHz. In order to increase this intrinsic bandwidth, one could use a smaller *T*_ϕ_, which, on the other hand, deteriorates the sensitivity. Similarly, according to the filter function of a dynamical decoupling sequence that has *n* number of π pulses,

(10) $$\begin{array}{l} G_{n}\left(\omega \right)=\frac{4{\left(\sin\frac{\omega T_{\phi }}{4n}\right)}^{2}\cos\left(\frac{\omega T_{\phi }}{2}-\frac{P\pi }{2}\right)}{\omega \cos\frac{\omega T_{\phi }}{2n}}, \end{array}$$

the bandwidth is determined by both *T*_ϕ_ and *n*. *P* is 0 when *n* is odd, and *P* is 1 when *n* is even. Figure 4A shows that the bandwidth becomes narrower when *n* grows larger. This property is used for nuclear magnetic resonance applications to achieve a high-frequency resolution (Aslam et al., 2017). However, the narrow bandwidth becomes a disadvantage for many other applications. Hybrid sequences can be used to extend the measurement bandwidth. For example, the Hahn-echo sequence can be combined with the CPMG-2 sequence. Given the signal frequency *f* = 1/*T*_ϕ_, the responses of the two sequences to a signal *B* sin(2π*ft* + φ) are *G*_1_ = 2*BT*_ϕ_ cos φ/π, *G*_2_ = 2*BT*_ϕ_ sin φ/π. The signal is resolved by calculating

(11) $$\begin{array}{l} B=\frac{\pi \sqrt{G_{1}^{2}+G_{2}^{2}}}{2T_{\phi }} \end{array}$$

(12) $$\begin{array}{l} \varphi =\arctan\frac{G_{2}}{G_{1}}. \end{array}$$

According to the new filter function $G_{12}=\sqrt{G_{1}^{2}+G_{2}^{2}}$, the 3-dB bandwidth can be calculated as *BW*_12_ ≈ 1.5/*T*_ϕ_, while the 3-dB bandwidth of the Hahn-echo sequence is *BW*_1_ ≈ 0.75/*T*_ϕ_. The bandwidth goes wider with shorter *T*_ϕ_, trading off with the sensitivity due to Equation (9). By concatenating sequences with different *T*_ϕ_, we can further extend the bandwidth but at the cost of a lower sampling rate. The sampling rate is determined by the total sequence length *T*_*seq*_. The inset of Figure 4B shows another concatenated sequence as an example, where we use two Hahn-echo sequences with *T*_ϕ1_ = 50 μs and *T*_ϕ2_ = 100 μs. As a result, the two Hahn-echo parts of the sequence cover the bandwidth of [6.25, 13.75] kHz and [12.5, 27.5] kHz respectively. Assuming that the two Hahn-echo parts of the sequence have the same sequence time, we get the sensitivity deterioration with a factor of $\sqrt{2}$.

Figure 4B further demonstrates the quantum phase signal responses measured in experiments regarding different sequences. Here we use the quantum phase sensitive detection (QPSD) technique described in the reference (Zhang et al., 2022), to acquire the quantum phase accumulated through the spin-field interaction. To characterize the frequency responses to different sequences, we apply “dc” fields (low-frequency oscillating fields) and ac fields with an amplitude of 500 nT. In the Ramsey measurement, *T*_ϕ_ = 5 μs and *T*_*seq*_ = 100 μs. The signal frequency response should be consistent with the filter function of the Ramsey sequence, which has a bandwidth approaching 100 kHz. However, the pulse sequence gives a much lower sampling rate due to the total time of one measurement sequence, which is 10 kHz. According to the Shannon theorem, the signal measurement bandwidth *BW* < 1/(2*T*_*seq*_) = 5 kHz, while we detect a drop of the acquired magnitude when the signal frequency approaches 5 kHz. Furthermore, we plot the signal responses of ac sensing schemes. In ac sensing schemes, *T*_ϕ_ of the sequence needs to be adjusted to get the best ac sensitivity according to the filter functions exampled in Figure 4A. According to Equation (10), it can be calculated that the signal response, i.e. the accumulated quantum phase, decreases when the signal frequency increases. By applying high-order dynamical decoupling sequences, the signal response increases with the order number as the arrow and note shown in the figure. Therefore, it is possible to concatenate dynamical decoupling sequences with different orders to achieve an ac field measurement with a wide bandwidth. The XY8 sequence is an alternate dynamical decoupling sequence that can reduce the influence of the pulse errors (Farfurnik et al., 2015). Usually, the highest detectable frequency is determined by the pulse width applied in the measurements, and the pulse width is determined by the finite MW power. Theoretically, the pulses are considered infinitely narrow. In experiments, when the pulse width is comparable to *T*_ϕ_, this pulse error can lead to a measurement failure. The XY8 sequence can suppress the influence of the pulse error and ensures the detection of high-frequency signals. In our measurements, the Hahn-echo sequence can measure a signal frequency of up to 1 MHz, while the XY8 sequence can measure a signal frequency of up to 5 MHz. Same to measurements with the Ramsey sequence, the bandwidth of the ac sensing scheme is also limited by the sampling rate, which is determined by the total sequence time. Although the high-frequency sensing characteristic is not that useful for bio-signal sensing applications, the corresponding dynamical decoupling techniques can also be used for low-kHz signal sensing by using NV ensembles with long *T*_2_. For example, with *T*_2_ = 200μs (Element Six, DNV-B1), the detectable frequency can be as low as 2.5 kHz. Even though, the frequency is still higher than the regular < 1 kHz bio-signals. Modulation techniques can be a solution to up-covert the dc field to a high-frequency ac field, and the ac magnetometry sequences can be applied to achieve *T*_2_ limited sensitivity (Wood et al., 2018; Xie et al., 2022). There are also cases that dynamical decoupling techniques, such as the WAHUHA sequence, are used to address the dipole-dipole interactions to extend $T_{2}^{*}$ and to improve the dc sensitivity (Balasubramanian et al., 2019).

To summarize the bandwidth results and discussions, the wide detectable frequency range of NV magnetometry in experiments is performed. The current bandwidth limit technically comes from the instrumentation and the low signal sampling frequency induced by measurement schemes. The concatenated sequence can be expected to extend the bandwidth at the cost of sensitivity. The lower limit of the detectable frequency can be extended by using NV ensembles with long *T*_2_ so that dynamical decoupling sequences can be used for bio-magnetic signal detection.

#### 2.2.3. Dynamic range

Dynamic range is another useful specification for magnetometry applications. However, there is usually a trade-off between the dynamic range and sensitivity of a sensor (Shah et al., 2010). For example, OPMs with fT sensitivity mostly require magnetic shielding to maintain the near-zero operation field range. OPMs that use a different scheme for extending the dynamic range to the geomagnetic level, compromise their dynamic ranges with pT sensitivity. On the other hand, NV magnetometry does not require a low-field environment to perform highly sensitive measurements; instead, it requires a bias field *B*_0_ that can range from geomagnetic level to a few Tesla (Stepanov et al., 2015). Therefore, NV magnetometry can perform measurements over a large magnetic field range. However, since the MW frequency applied in NV magnetometry significantly changes with *B*_0_, the dynamic range of an NV sensor is usually limited by instrumentation. Phase-estimation-algorithm (PEA) and QPSD technique can be used to extend the dynamic range of NV sensors based on interferometry schemes (Nusran et al., 2012; Zhang et al., 2022). Nevertheless, the MW frequency should be near resonant to the NV spins, which leads to a linewidth of a few MHz, corresponding to a dynamic range of roughly 1 Gauss. The frequency-locking feedback technique can further extend the dynamic range of NV magnetometers by tracking the resonant frequency (Clevenson et al., 2018). In principle, the dynamic range can be as large as the operable *B*_0_ range regardless of the sensitivity.

**Figure 5:**
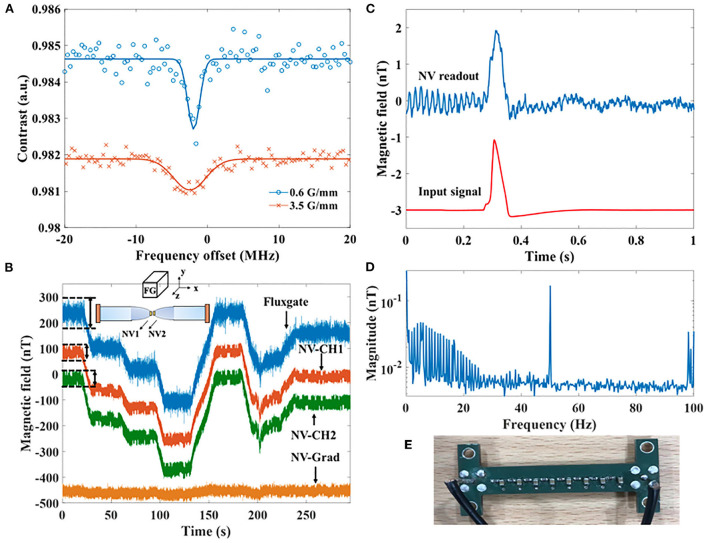
**(A)** ODMR spectra with different local gradients at the diamond. **(B)** Magnetic field detected by the NV gradiometer in the unshielded environment with the comparison of a fluxgate magnetometer. The schematic of the setup is shown as the inset drawing. The step signals are generated by an elevator nearby. From top to bottom, the lines are signals from the fluxgate sensor, NV gradiometer channel 1 and channel 2, and the output of the NV gradiometer, i.e., subtraction of the two channels. The dashed lines and arrows are the eye-guide for comparing the noise of NV channels and the fluxgate magnetometer. **(C)** The action potential signal (generated by a phantom) detected by the NV magnetometer. The original input of the action potential is as the lower waveform shows. The detected magnetic field amplitude is about 2 nT. **(D)** is the Fourier transform of the detected action potential signal. The principle noise peaks are at dc, 50 Hz, and 100 Hz. **(E)** The phantom used for generating magnetic field signals to simulate the MMG signal.

In addition, we note that the sensitivity of NV sensors may deteriorate under a high *B*_0_ field. The trade-off origins from the fact that a high *B*_0_ gradient can be induced by the high field. In the experiment, the diamond has a dimension of 1 mm, and the dephasing time $T_{2}^{*}=3.4\text{ μs}$ is measured under a field *B*_0_ ≈ 10 G. In order to see the deterioration of the sensitivity due to a large gradient, we put the sensor at different locations in a pair of permanent magnets so that the ODMR spectrum is measured under different gradients, as shown in Figure 5A. With a higher gradient, the ODMR linewidth is significantly broader, and the contrast is smaller, which leads to the deterioration of sensitivity according to Equation (2). For the NV sensors with pT sensitivity, the required linewidth is a few hundred kHz which corresponds to the magnetic field gradient < 0.1 G/mm. Therefore, although the dynamic range of NV sensors can be very large, it is still preferred to operate the sensor in a normal environment without a large magnetic gradient over the diamond.

### 2.3. Gradiometry and pseudo-signal detection

With all the specifications discussed above, one of the expectations of NV sensors is to achieve gradiometry measurements of bio-signals in an unshielded environment. Without magnetic shieldings, magnetic noises from sources that are far away from the gradiometer channels will be detected as common mode noises. As a result, the signal-noise ratio can be improved by using NV gradiometer without shielding. Figure 5B presents the time traces measured by the NV gradiometer with the result of a fluxgate magnetometer as the benchmark. From the time traces of each individual channel, one can see the step responses, which is the noise generated by an elevator roughly 10 m away. The elevator was controlled to go up from the ground floor and stop at the 3rd, 6th, and 9th floor, respectively, and then the elevator went down directly to the ground floor at around 200 s. We place two NV probes as Figure 1C with their diamonds 5 mm away from each other. The readout of the two probes are calibrated by known fields generated by a Golay coil (see Supplementary Note 2). A fluxgate magnetometer is placed 10 cm above the two NV channels with its *z* direction aligning along the NV orientation of the two diamonds. The inset of Figure 5B shows the schematic of the setup. Since we do not have an identical diamond to the one with $T_{2}^{*}=8.5\text{ μs}$, we use another pair of (111) diamonds cut from a plate. The diamonds used for the gradiometer channels are two 1 × 1 × 0.5 mm^3^ diamonds, which are isotopic purified and include NV center ensembles with $T_{2}^{*}=3.4\text{μs}$. The signal read out from the fluxgate magnetometer is used as a reference to see if the gradiometer measures magnetic field noise correctly. Because of the 100 mW laser power, the CW-ODMR scheme is used for signal detection. Hyperfine driving and double resonance driving are used to enhance the sensitivity (Fescenko et al., 2020; Zhang et al., 2021). The figure shows that the NV channels pick up the same signal as the fluxgate magnetometer. With the gradiometry scheme, the noise introduced by the elevator is removed as the time trace at bottom of the figure. By suppressing this common mode noise, the gradiometer makes it possible to resolve small signals from the result without the fluctuation of hundreds of nT. The residual noise shown in the gradiometry trace is majorly induced by the 50 Hz harmonics. In order to have a comparison between the time traces of the NV gradiometer channels and the fluxgate sensor, we use the dashed lines and arrows in Figure 5B as the eye-guide. It can be straightforwardly seen that the NV channels are less noisy than the output of the fluxgate magnetometer. However, the unshielded gradient noise is still so large that the intrinsic noise of the gradiometer cannot be shown in such a measurement. Therefore, we measures the laser noise (magnetic insensitive) to label the sensitivity of the gradiometer, which is 39–46 $\text{pT}/\sqrt{\text{Hz}}$ (see Supplementary Figure 2).

In order to investigate how sensitive the NV sensors can be, we perform a measurement of simulated action potential signal inside of a shielding using the diamond with $T_{2}^{*}=8.5\text{ μs}$, as shown in Figures 5C, D. The signal is generated by an arbitrary waveform generator and sent to the phantom as Figure 5E shows. The phantom is designed to simulate the triggered electric signal in muscle fibers. The phantom is placed 1 cm below the diamond, and the signal is applied repeatedly for 60 s. We cut the output time trace measured by the NV magnetometer into 1 s intervals and average the intervals so that the waveform can be compared to the input signal. Figure 5C shows that the NV readout recovers the action potential reasonably from harmonic noises during the measurement time. Figure 5D shows the signal spectrum, and the noise level is shown with the 60 s measurement. According to the spectrum, the noise floor is around 2–3 pT, corresponding to a sensitivity of 15.5–23.2 $\text{pT}/\sqrt{\text{Hz}}$. The noise increases to 100 pT near DC due to the 1/f noise. Besides, the electrical harmonics still have a level of 100 pT after the attenuation of the μ-metal shielding from roughly 10 nT. The magnitude of the measured magnetic field pulse is about 2 nT, while the maximal magnitude of the signal components is only 50 pT. This measurement shows the possibility of detecting an action potential waveform with pT sensitivity.

## 3. Potential bio-magnetic field detecting applications for NV magnetometry

### 3.1. Magnetoneurography

**Figure 6:**
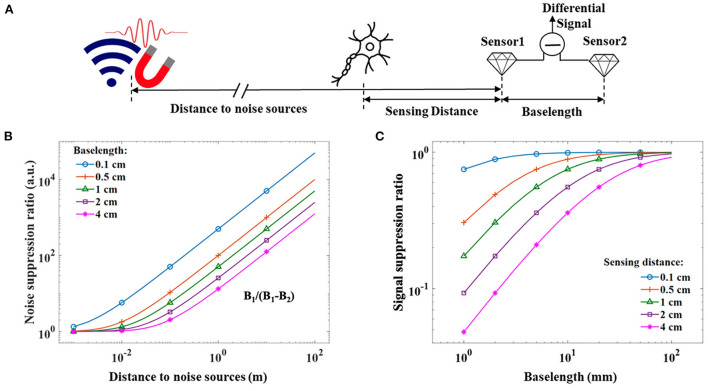
**(A)** Sketch of relevant parameters that have to be taken into account when compromising the noise and signal suppression in a linear gradiometer configuration. **(B)** Noise suppression ratio for different baselengths as a function of the distance to the noise sources. **(C)** Relative differential signal of the gradiometers for different distances to the signal source as a function of baselength. For both cases, it is assumed that the magnetic field strength of the noise source and signal sources decay as *B*(*r*) ∝ *r*^−2^ with distance to the different sources.

As stated in the introduction, one of the most important benefits that NV magnetometers can get is reducing the sensing distance from the sensor to the sensing target. In this section, we discuss the potential application of MNG and the idea of using NV magnetometers to improve the signal-noise ratio of MNG. Normally, MNG detection with non-invasive techniques is very challenging as magnetic fields from the nerve bundles require $\text{fT}/\sqrt{\text{Hz}}$ sensitivity if measured a few or tens of millimeters away from the human body (Murzin et al., 2020). The decrease of the magnetic fields with increasing distance from single nerves has been investigated theoretically and experimentally and is dependent on the length of the fiber and the distance itself (Williamson and Hoke, 2012). MNG signals are usually superpositions from the simultaneous activation of many nerve action potentials and the accompanying ionic flow that causes radiation of magnetic compound action fields (MCAFs). The dispersion effect of the nerve conduction velocity leads to a quadratic distance dependence of the magnetic fields (Trahms et al., 1989). The ability of NV-magnetometers to provide high sensitivity in a small sensing volume enables the possibility to position the sensor in the vicinity of a nerve structure so that the requirement for the highest sensitivity can be reduced. For example, this is the case for potential intraoperative applications, where nerve structures can be approached within a few or even < 1 mm. Thus, magnetic contactless functional testing of nerves or the localization of nerve bundles during surgery may become possible. Areas of intraoperative neuromonitoring which could benefit from such a technology are, e.g., the localization of critical nerve bundles such as the facial nerve in cochlea implant surgery or during brain tumor resection (Heman-Ackah et al., 2013; Schucht et al., 2015). Currently, intraoperative monitoring of these structures is usually done by electrical stimulation techniques that suffer from patient-specific differences in tissue morphology and tissue impedance (Ansó et al., 2019). The main challenge for the magnetic detection of bio-magnetic fields in such scenarios is the discrimination of magnetic signals from other sources that are present in a surgery room. Such noise sources comprise but are not limited to, power lines, electrical medical equipment, moving metallic parts such as surgical instruments, or further bio-magnetic signals beyond the specific signal of interest. The main parameters which dictate the noise suppression of a gradiometer are the distance to the noise source, the distance to the object of interest (sensing distance) as well as the baselength of the two sensors in the gradiometer as well as the orientation of all devices and thus orientations of involved magnetic field components. Figure 6 sketches the relevant parameters for potential intraoperative MNG measurements, where all components are oriented along a single axis for simplification. A noise suppression ratio of a gradiometer can be defined as the quotient of the magnetic field that a magnetometer would detect from a noise source and the remaining magnetic field noise that a gradiometer would still detect from the same noise source:

(13) $$\begin{array}{l} SR_{noise}\left(b,r\right)=\frac{n_{sig}\left(r\right)}{n_{grad}\left(b,r\right)}=\frac{B_{1}}{B_{1}-B_{2}}, \end{array}$$

where *SR*_*noise*_ is the noise suppression ratio, *n*_*sig*_ is the noise detected by a single channel magnetometer and *n*_*grad*_ is the gradient noise. The noise suppression ratio strongly depends on the distance of the sensor to the noise source *r* and the baselength *b*. An exemplary estimation of a noise suppression ability for noise sources whose magnetic field strength decay with distance according to *B*(*r*) ∝ *r*^−2^ is depicted in Figure 6B. A small baselength is preferred for high noise suppression but may lead to an unintentional suppression of the signal of interest as well. The level of signal suppression for specific baselength configurations can be evaluated in analogy to the noise suppression. The signal suppression ratio is then given by the quotient of the diminished signal that a gradiometer would detect, normalized to the signal that a single channel magnetometer would detect from the source of interest (e.g., from a nerve bundle):

(14) $$\begin{array}{l} SR_{signal}\left(b,r\right)=\frac{m_{grad}\left(b,r\right)}{m_{sig}\left(r\right)}=\frac{B_{1}-B_{2}}{B_{1}}, \end{array}$$

where *SR*_*signal*_ is the signal suppression ratio, *m*_*grad*_ is the magnetic field that the gradiometer detects from the object of interest, and *m*_*sig*_ is the magnetic field detected by a single channel magnetometer from the same object. Figure 6C depicts the relative signal difference for gradiometers with various distances from the object of interest as a function of baselength, assuming a quadratic distance dependence of the magnetic field again. As a small baselength can strongly suppress the signal of interest, the application, and environment define the optimal baselength to achieve the highest possible signal-to-noise ratio. As a reasonable trade-off that avoids a signal suppression by more than 25% and still maintains a good noise suppression, a baselength as long as the application specifically expected sensing distance could be chosen. Ideally, gradiometers are designed with flexible baselengths, which has been realized recently with fiber-coupled NV-magnetometers (Masuyama et al., 2021).

### 3.2. Magnetonmyography

Instead of neuron fiber bundles, MMG measures magnetic field signals that are triggered along the muscle fiber bundles. Therefore, similar to MNG, NV magnetometers can be used to improve the SNR by approaching closer to muscle fiber bundles. Moreover, the largest MMG signal can be hundreds of pT above the skin, which makes it more promising than detecting MNG signals by NV magnetometers. In this section, we describe the expectations of using MMG with NV magnetometry as a future diagnostic tool.

Skeletal muscle contraction is controlled by the nervous system (Kandel et al., 2000; Heckman and Enoka, 2012) through electric signals (MacIntosh et al., 2006; Röhrle et al., 2019), i.e., propagating muscle fiber action potentials, yielding electromagnetic fields. Hence, measuring muscle-induced electromagnetic fields can provide information on the neural control signals to the muscle as well as the electrophysiological function of the muscle itself. Thereby, one distinguishes electromyography (EMG) (cf. e.g., De Luca, 1997; Merletti and Farina, 2016), which measures muscle-induced electrical potential, and MMG, i.e., recording the muscle-induced magnetic field. Several physical considerations regarding the properties of muscle-induced bioelectromagnetic fields motivate the development and investigation of MMG technology. Considering well-established EMG measurements, signals can be measured invasively *via* needle or fine-wire electrodes (Merletti and Farina, 2009) or by means of non-invasive surface electrodes (Farina et al., 2014). Signals obtained from needle EMG recordings are typically easy to interpret but only represent the activity of a handful of active motor neurons in the spinal cord. Moreover, the invasive nature of the measurements limits the application of intramuscular EMG to collaborative patients or subjects. Surface EMG, on the other hand, is strongly influenced by the electric tissue properties, i.e., acting as a low-pass filter (Roeleveld et al., 1997; Lowery et al., 2002; Klotz et al., 2020), and hence, the signal provides a more global view of the muscle. This comes with the cost that the interpretation of surface EMG is more challenging and the results of surface EMG-based studies are often subject to considerable uncertainties. Unlike electrical potentials, magnetic fields can be propagated through the body without distortion (Malmivuo et al., 1995; Oschman, 2002). A recent simulation study (Klotz et al., 2022) shows that MMG signals are less affected by subcutaneous fat than a corresponding EMG signal. Accordingly, non-invasive MMG is superior to EMG for distinguishing spatially shifted muscle fiber sources; something highly desirable, for example, for decomposing an interference signal into the spike trains of individual motor units (e.g., Holobar and Zazula, 2007; De Luca et al., 2015; Negro et al., 2016) and for detecting hallmarks of neuromuscular disorders (e.g., Rubin, 2019; Marquetand et al., 2021). However, whether MMG provides any advantage over EMG for obtaining novel insights into the neuromuscular system has yet to be explored experimentally. This requires novel MMG detection systems, particularly with the capability to sample the muscle-induced magnetic field *via* dense arrays of sensors with a small detection volume, e.g., NV magnetometers. Using a dense array of MMG sensors, i.e., high-density MMG, would also allow performing spatial filtering (the most intuitive way to think of spatial filtering is to picture the function of an operational amplifier in common-mode rejection). This technique is routinely used in electromyography to enhance the signal components associated with muscle fibers active in the proximity of the sensors and allows to drastically reduce the influence of background noise.

**Figure 7:**
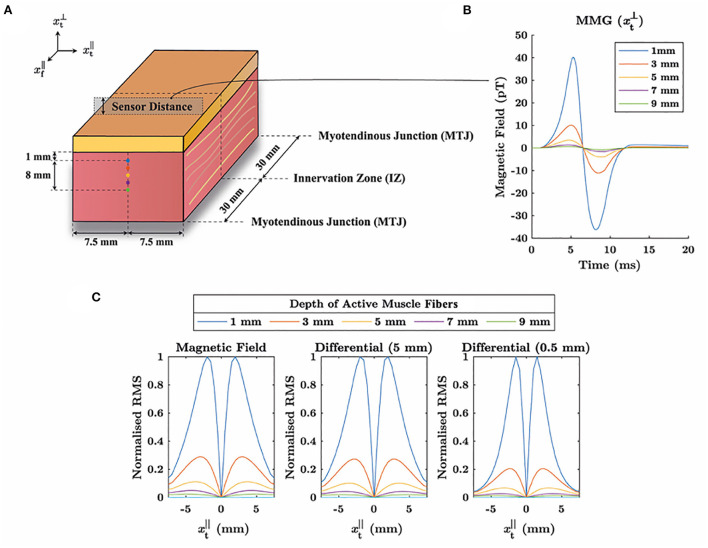
**(A)** For a cube-shaped muscle compound, muscle action potentials are simulated when muscle fibers in different depths are stimulated. A 2 mm thick fat tissue layer is added on top of the muscle, and the magnetic field is observed midway between the innervation zone (IZ) and the myotendinous junction (MTJ). For obtaining a differential signal, the magnetic field is sampled at the body surface and in a line with distance $\Delta x_{\text{t}}^{⊥}$ above the muscle surface. **(B)** Exemplary MMG signal measured at one point with a distance of 0.5 mm to the body surface when varying the depth of the active muscle fibers. **(C)** Signal amplitude on the body surface in a line perpendicular to the muscle fibers (cf. **A**). The left column shows the root mean square (RMS) of the raw MMG signal, depending on the spatial coordinate and the depth of the active muscle fibers. The middle and the right column show the RMS distribution corresponding to differential signals with inter-sensor distances of 5 and 0.5 mm, respectively. Thereby, it can be observed that decreasing the distance between the sensors narrows the amplitude distributions and hence reduces the detection volume of measurement.

Figure 7 showcases that differential recording allows to further increase in the spatial sensitivity of MMG measurements. Therefore, using a computational model (Klotz et al., 2022), muscle contraction is simulated by selectively stimulating muscle fibers in different depths. The virtual MMG is sampled midway between the innervation zone and the myotendinous junction on the muscle surface and at a distance of $\Delta x_{\text{t}}^{⊥}$ from the muscle surface. The geometry of the muscle and the basic simulation set-up is shown in Figure 7A. Further, Figure 7B shows the time-domain MMG signal at one exemplary selected sampling point. It can be observed from Figure 7C that the spatial sensitivity of a differential recording is correlated with the inter-sensor distance, i.e., decreasing the distance between the sampling points increases the spatial sensitivity. That is, the amplitude decay with increasing fiber depth is more pronounced. In detail, comparing the signal amplitude for the simulation with a muscle fiber depth of 3 mm to the simulation with a fiber depth of 1 mm, the RMS (root mean square) value is 29.0% for the raw MMG data, 27.3% for $\Delta x_{\text{t}}^{⊥}=5\text{mm}$ and 20.5% for $\Delta x_{\text{t}}^{⊥}=0.5\text{mm}$. Further, the peak width decreases when reducing the vertical spacing of the sampling points. For example, for a fiber depth of 1 mm values larger than 50% of the maximum can be observed along a line with a length of 4 mm for the raw MMG data, 3.5 mm for $\Delta x_{\text{t}}^{⊥}=5\text{mm}$ and 3 mm for $\Delta x_{\text{t}}^{⊥}=0.5\text{mm}$. This example indicates the expected parameters that should be used in such an MMG measurement, where at least a sub-cm spatial resolution is required. NV magnetometry, accordingly, is a perfect candidate for the technique, as it is possible to have the sensor head with a dimension of a few millimeters.

## 4. Conclusions

From the perspective of bio-sensing applications, the performance of NV magnetometry is comprehensively presented in this work. In this work, we analyze the sensitivity, bandwidth, and dynamic range for both the ODMR sensing scheme and the interferometry sensing schemes. NV magnetometers are able to show sensitivities of pT level to even sub-pT level, and they can measure signals up to MHz with extended bandwidth by using concatenated sequences. We briefly discussed the extension of the dynamic range of NV magnetometry, and point out the issue of deteriorated sensitivity due to gradients. Experiments of measuring magnetic field signals by both magnetometer and gradiometer are demonstrated to show the performance of the NV sensors, where we see the capability of detecting a simulated action potential signal and suppressing environmental noise by the gradiometer. Finally, discussions regarding the potential applications, i.e., MNG and MMG, are elaborated for understanding the benefits of introducing NV magnetometry to the studies. With the discussions, we hope that NV magnetometers can be further developed for detecting either MNG signals or MMG signals in the next stage.

## Data availability statement

The raw data supporting the conclusions of this article will be made available by the authors, without undue reservation.

## Author contributions

The original draft was completed by the joint efforts of CZ, JZ, MW, TK, LG, and PB. CZ, JZ, and MW prepared Sections 1 and 2. TK and LG prepared Section 3.1. PB prepared Section 3.2. JW and DD helped review and edit the article. CZ performed the experiments with the assistance of MB and MK. The work was supported and supervised by JW and OR. All authors contributed to the article and approved the submitted version.
