# Supplementary material for: Optimizing NV magnetometry for Magnetoneurography and Magnetomyography applications
Source: Front Neurosci. 2023 Jan 12;16:1034391. doi: 10.3389/fnins.2022.1034391 (PMC9885266; doi:10.3389/fnins.2022.1034391)
Supplement: Supplementary file 1 [file Presentation_1.PDF]

Supplementary Material

1 SUPPLEMENTARY NOTES

1.1 Note 1: Estimation of sensitivity enhancement with laser saturation

In the experiment, the laser power is far lower than the saturation power. This causes two issues, i) the fluorescence photon number per measurement is smaller than the optimized value; ii) the slow repolarization process introduces a long head time in the sequence. Although the optimized field integration time in the experiment is about 6.4 μs, the measurement sequence is 500 μs to ensure a complete repolarization of the spin ensemble. With the $(0.5 \text{ mm})^3$ cube diamond and $[N] = 0.4 \text{ ppm}$, the number of NV centers used in the diamond is estimated as 8.8×10^{12}. However, the photon count per measurement in the experiment is only 4.6×10^{10}. Therefore, by saturating the laser power, the photon count per measurement can at least enhance by 100 times. On the other hand, the head time for repolarization can be shortened to roughly $1 \ \mu$s, which decreases the sequence time by a factor of $500/(6.4 + 1) \approx 67$. Since $\eta \propto \sqrt{T_{seq}/\mathcal{N}}$, the sensitivity can be enhanced by a factor of ≈ 110, i.e. 155 $\text{fT}/\sqrt{\text{Hz}}$ from the measured 17 $\text{pT}/\sqrt{\text{Hz}}$. There is another factor of 2 from double quantum magnetometry that the estimation can take into account. Thus, a $< 100 \ \text{fT}\sqrt{\text{Hz}}$ sensitivity can even be expected. Nevertheless, the Watts level laser power is a huge obstacle to applications. It not only introduces extra noise due to heating but also has potential hazards to subjects.

1.2 Note 2: Calibration of the gradiometer

The NV gradiometer used in the work is calibrated by known signals generated from coils. A Golay coil is used to generate a gradient field for the two channels. For the first step, the ODMR spectra of the two channels are measured with different currents in the coil. When the current is zero, the splitting of the ODMR spectrum is set as the reference for identifying the splitting changes. Since the splitting only depends on the external field and the electron gyromagnetic ratio, the ODMR spectrum can be used to calibrate the coil. Through the splitting changes of the transition lines in each ODMR spectrum, the magnetic fields at each channel are determined by calculating Δ/γ_e, where Δ is the splitting frequency and $\gamma_e = 28\text{Hz}/\text{nT}$ is the electron gyromagnetic ratio. The coefficients of the Golay coils at the two diamond spots are calibrated by fitting the plots, as shown in figure S2. Then, the coil is used to send an oscillation field (at 70 Hz) according to the calibrated results, and the field calibrates the photodetector readout of the NV magnetometer with fixed MW frequencies. The scalar factor of the NV magnetometer is acquired by comparing the readout magnitude to the field magnitude, which are 9.95×10^{-7} V/nT and 1.16×10^{-6} V/nT, respectively. The two scalar factors are smaller than the scalar factor measured with the magnetometer inside of a shielding, which is 3×10^{-6} V/nT. The difference mainly comes from the T_2^* of the different diamonds. With the scalar factors, The equivalent unshielded gradient noise limit and the shielded magnetic noise limit are measured with the laser on while the MW is off-resonance to the sensors.

2 SUPPLEMENTARY FIGURES

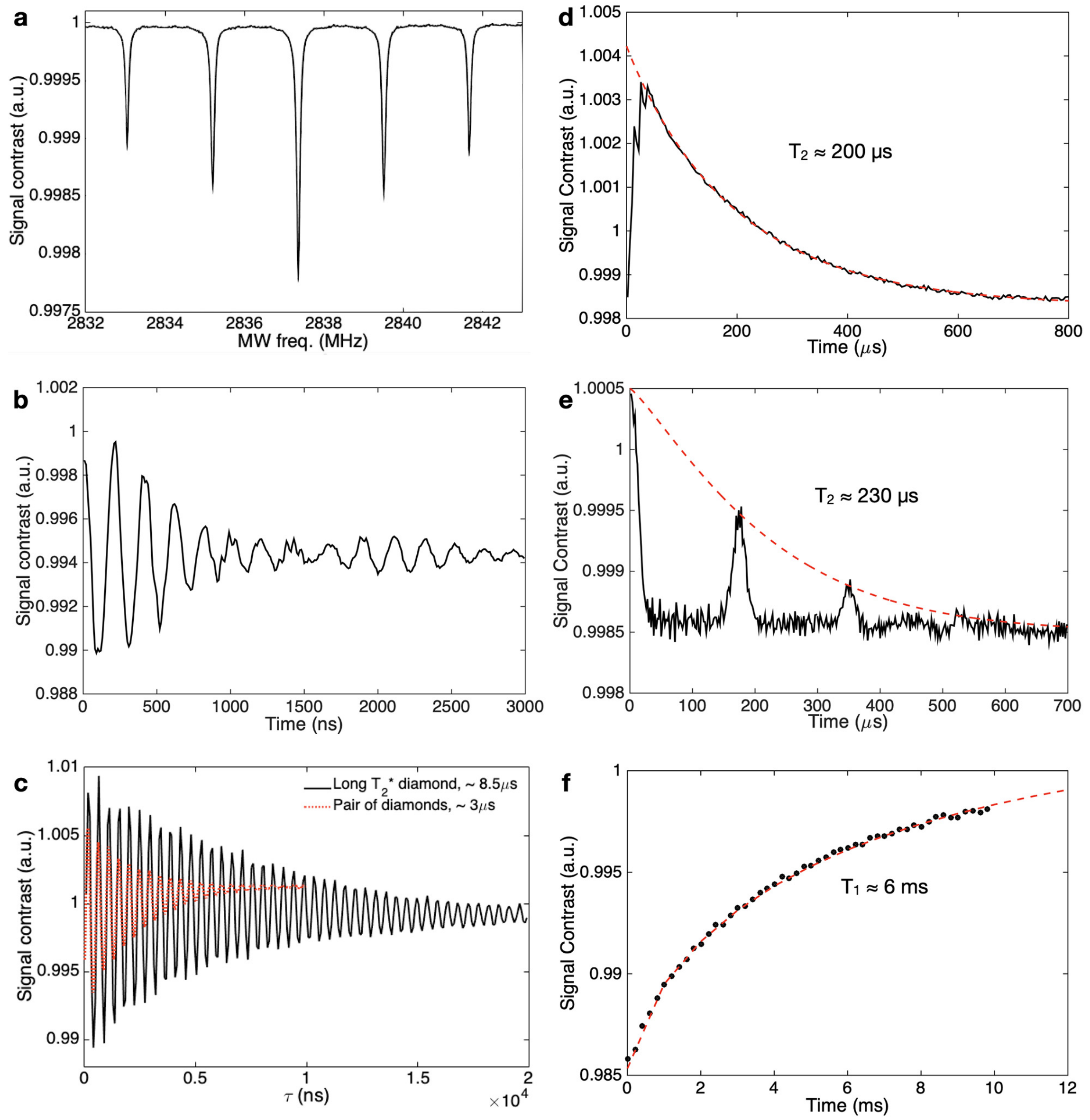

Figure S1. (a). The CW-ODMR spectrum of the transition lines $|0\rangle \rightarrow |-1\rangle$. There are five lines because three MW frequencies are used to drive the three hyperfine lines ^{14}N nuclei spin, i.e. hyperfine driving. (b) Rabi oscillation in experiments. The Rabi frequency is 5 MHz. (c) The T_2^* measurements with Ramsey sequence. The diamond is an isotopically purified (0.5 mm)^3 diamond and a pair of $1 \times 1 \times 0.5\text{mm}^3$. (d) The measured $T_2 \approx 200$ μs with the same diamond used in c. (e) The measured $T_2 \approx 230$ μs with the diamond DNV-B1 from Element Six. There are revivals due to the ^{13}C nuclear spins in the diamond. As a result, the ac signals can only be measured with the measurement times when there are revival peaks. (f) The measured $T_1 = 6$ ms with the diamond used in c and d.

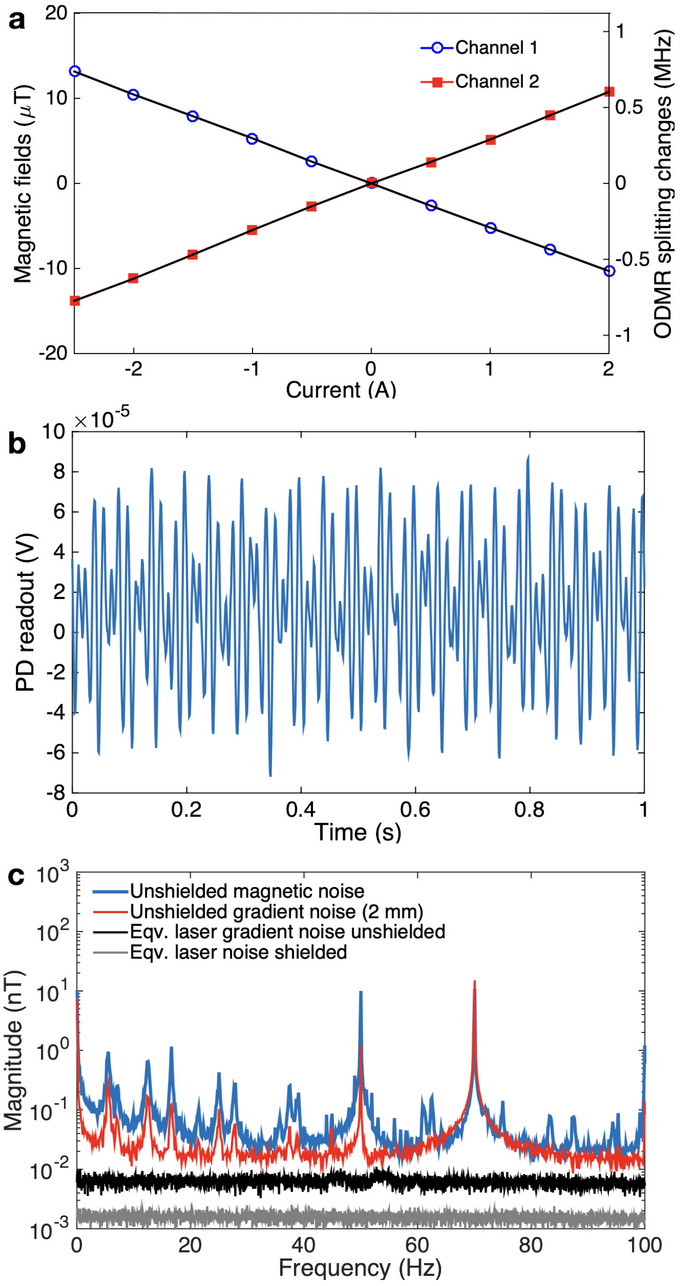

Figure S2. (a) Calibration of the Golay coil with the ODMR splitting of the two diamond sensor channels. Currents are sent to the coil and generate a decreasing field at channel 1 and an increasing field at channel 2. The magnetic fields are determined as -5.22 $\mu\mathrm{T}/\mathrm{A}$ at channel 1, and 5.45 $\mu\mathrm{T}/\mathrm{A}$ at channel 2. (b) The time trace of the photodetector readout in presence of a 70 Hz calibration field and the magnetic noises of the environment. (c) The magnetic noise spectra are measured by the unshielded gradiometer, and the laser noise (calculated as magnetic fields) is measured by the magnetometer inside/outside a shield. The blue spectrum is the magnetic noise spectrum measured by a single channel. The red one is the gradient spectrum acquired by subtracting the time traces of the two channels. The black spectrum is the equivalent laser noise acquired by the gradiometer outside of the shielding, which is higher than the laser noise measured by the shielded magnetometer (in grey) due to the longer T_2^* of the diamond used in the shielding.

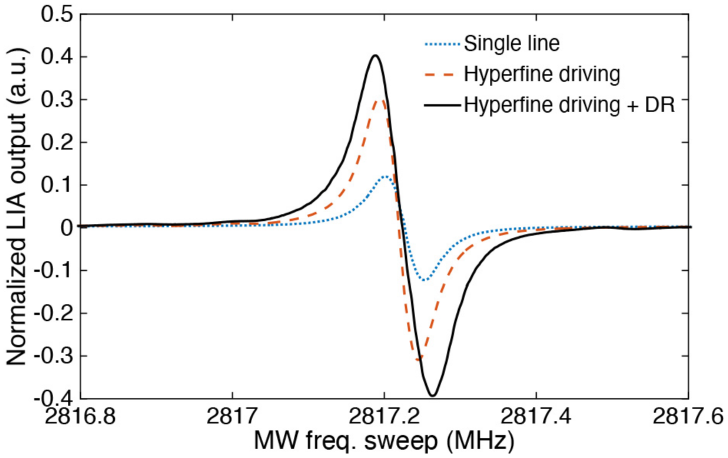

Figure S3. Enhancement of CW-ODMR signal contrast with hyperfine driving and double resonance driving. The ODMR spectra are acquired from the LIA output and are normalized to see the contrast difference. The blue dotted curve shows the spectrum with single MW component. The red dashed curve shows the spectrum with three MW frequencies to drive all the hyperfine lines. The black solid line is the spectrum acquired from the measurement that utilizes both hyperfine driving and double resonance driving.
